# The psychological experience of body image change in adult stoma patients: A systematic review and qualitative meta‐synthesis

**DOI:** 10.1111/bjhp.70113

**Published:** 2026-10-12

**Authors:** Siân Levell, Sophie Buckley, Michael Baliousis

**Affiliations:** ^1^ School of Medicine University of Nottingham, Queen's Medical Centre Nottingham UK; ^2^ School of Psychology University of Lincoln Lincoln UK; ^3^ Cancer Psychology Service Nottingham University Hospitals NHS Trust Nottingham UK

**Keywords:** body image, meta‐synthesis, psychological experience, stoma, systematic review

## Abstract

**Purpose:**

Previous research demonstrates that body image concerns are highly prevalent in stoma patients and can significantly limit quality of life, yet the psychological processes underlying these changes remain under‐theorized in the literature. This review aims to develop an understanding of the psychological experience of body image change in adult stoma patients.

**Methods:**

A systematic review and meta‐ethnography was undertaken of primary, qualitative, English‐language research focusing on the psychological experience of body image change in adult stoma patients. Eight hundred and forty‐three studies were initially obtained from backwards citation chaining and three databases: Medline, CINAHL and PsychINFO. Methodological quality and risk of bias were evaluated using the Joanna Briggs Institute Qualitative Checklist.

**Results:**

After screening, 18 studies (*n* = 602) were included in the synthesis. Three third‐order constructs were found across two time phases (immediately after surgery, and living with the stoma): all‐consuming shock, visible ‘otherness’ and fluctuating acceptance. Findings map the trajectory of body image change following stoma formation and identify psychological, social and relational processes associated with continuing distress and adaptation.

**Conclusions:**

This review moves beyond documenting body image distress in stoma patients to explain the psychological mechanisms through which it develops and fluctuates over time. Stoma formation can disrupt bodily integrity and identity, with distress maintained through self‐scrutiny, shame and perceived social exposure. The mechanisms identified provide clear targets for tailored psychosocial support beginning pre‐surgery and continuing through recovery to reduce long‐term body image distress and improve health‐related quality of life.

**Statement of Contribution**

**What is already known on this subject?**

- Body image concerns are prevalent in people with stomas and are linked to reduced quality of life.
- Many struggle to integrate their changed body with their mental representation of the self.
- Living with a stoma creates unique body image challenges beyond other treatments like surgery.

**What does this study add?**

- Reconciliation of the bodily self is a continuous process which occurs across the initial recovery period and beyond
- Acceptance fluctuated over time and context and was supported by psychological distancing and validation from close relationships and valued activities.
- Shame, stoma visibility and internalization of body image standards are key drivers of distress.

## INTRODUCTION

A stoma is a permanent or temporary opening in the abdomen used to allow passage of bodily waste when parts of the digestive system have been bypassed or removed. Around 177,000 people in the United Kingdom live with a stoma, and a further 21,000 undergo stoma formation each year (Kettle, 2019). Procedures include ileostomies, colostomies and urostomies, which divert waste through the ileum, colon or bladder. Over half (55.7%) of stoma surgery is performed in the management of colorectal cancer and inflammatory bowel disease, and for many, this procedure can be lifesaving (Jansen et al., 2015; Mitchell et al., 2020). However, stoma‐related complications are common in up to 92% of patients, ranging from concerns with leakage or bleeding to reduced quality of life and high rates of psychological distress (Alenezi et al., 2021; Farahani et al., 2022; Kovoor et al., 2023; Malik et al., 2018; Rafiei et al., 2017; Reese et al., 2018). As such, recovery from stoma surgery can be complicated and often involves prolonged physical and psychological adjustment to the profound changes in appearance and functioning (Claessens et al., 2015; Kittscha et al., 2022; Zhang et al., 2019).

Body image, defined as a person's subjective thoughts, feelings and perception of their appearance, is widely cited across the literature as a critical driver of psychosocial distress for individuals with stomas (Alenezi et al., 2021; Ayaz‐Alkaya, 2019). Appearance changes after stoma surgery are vast and can include the protrusion of the stoma from the abdominal wall, the visibility of the stoma bag under clothes, scars or colour and texture changes in the surrounding tissue (Crohn's and Colitis UK, 2025). The impact of stoma formation on bodily functioning can significantly disrupt a person's integration of their altered body and sense of self; individuals describe themselves as disfigured following stoma formation and express a helplessness which can impact social functioning, employment and sexuality (Alenezi et al., 2021; Thorpe et al., 2009, 2016). Empirical evidence demonstrates that dissatisfaction with body image following stoma surgery significantly mediates the relationship between stoma status and quality of life (Silva et al., 2020; Song et al., 2020). This can have harmful consequences for self‐management of the stoma, creating a cyclical process where poor management exacerbates psychological distress, further undermining self‐care in turn (Altuntas et al., 2012; Liao & Qin, 2014; Lim et al., 2015; Pittman et al., 2008; Zhang et al., 2019). Despite this, body image needs are often overlooked in approaches to psychological support for stoma patients, which may explain why psychological difficulties in this area persist despite overall improvements in a patient's quality of life (Noone, 2010; Reese et al., 2018).

The experience of body image change following appearance‐altering conditions is acknowledged to be psychological in nature, consisting of internal mental processes like cognition, perception and affect (Melissant et al., 2021; Rhoten, 2016; White, 2000). Previous research into the psychological experience of body image change indicates that the defining attributes of this are: perceived appearance changes, internalization of body image ideals, automatic thoughts and assumptions and levels of emotional distress (e.g., shame and fear) (Melissant et al., 2021; Rhoten, 2016; White, 2000). While these factors have provided a useful framework for understanding the phenomena of body image change in other areas, the availability of research with stoma populations is minimal. Individuals with stomas report poorer body image than those who have experienced alternative treatments like surgery, and the addition of stressors like clothing restrictions, leakage and visibility of the stoma may add a unique element to a person's body image appraisal (Osborne et al., 2022; Sharpe et al., 2011). Despite this, research into the psychological experience of body image change in stoma patients has been inclined to focus primarily on clinical outcomes or psychological predictors such as depression, anxiety or quality of life (e.g., Kovoor et al., 2023).

A deeper understanding of the psychological mechanisms underlying psychological adjustment to body image change in stoma patients is needed to inform targeted interventions that prevent or reduce distress and promote positive adaptation. Quantitative research tells us that factors like self‐reported well‐being and resilience can predict body image outcomes, however, it does not tell us how the varying individual, social and health care circumstances constitute this change, therefore indicating a gap (Guo et al., 2025; Sarah et al., 2025). Synthesizing findings from qualitative research can provide an initial understanding of these mechanisms and processes, guiding the development of psychologically informed interventions and their future validation through quantitative research. This review therefore aims to synthesize qualitative evidence on the psychological processes and lived experience of body image change in adults with stomas.

## METHODS

### Protocol registration

This review was registered prospectively on PROSPERO and adheres to the Preferred Reporting Items for Systematic Reviews and Meta‐Analyses (PRISMA) guidelines (registration number: CRD420251087477, protocol available at: https://www.crd.york.ac.uk/PROSPEROFILES/8908abe90437d4d9c750ec5adc0b1f0f.pdf). No changes were made to the eligibility criteria, search, screening or synthesis methods specified at registration. Following peer review, GRADE‐CERQual was added to assess confidence in each review finding; this post‐protocol addition did not alter study inclusion or the synthesis findings.

### Study design

A qualitative meta‐synthesis was carried out under a critical realist epistemology. Critical realism posits that reality exists independently of our perceptions, but that our knowledge of reality is mediated through social, cultural and linguistic frameworks (Bhaskar, 2020). This perspective suits the aims of the review; while stoma formation involves observable physical change, the *meaning* of that change is constructed through personal, cultural and contextual factors. Adopting a critical realist stance therefore allows exploration of how individuals' appraisals of their bodies interact with the social structures that shape those appraisals (Vincent & O'Mahoney, 2018).

### Eligibility

Eligibility was defined using the PICo framework (Hosseini et al., 2024):

- Population: Individuals aged 18 and over with a stoma. Previous research indicates that body image differs between adults and under 18 s, with adolescence characterized by ongoing physical, psychological and identity development that contributes to greater fluctuation in body image (Bellard et al., 2021; León et al., 2021; Quittkat et al., 2019). Including only adult populations in this review therefore enabled the synthesis to focus on body image changes associated with stoma surgery, rather than normative developmental changes. Furthermore, no restrictions were placed on gender, as including participants of all genders captured a broader range of experiences and permitted consideration of any gender‐related differences reported within the primary studies.
- Phenomena of Interest: Psychological processes and lived experience of body image change after stoma surgery, where psychological experiences were conceptualized as consisting of internal, mental processes
- Context: Any clinical, community, cultural or geographical context in which adults live with or adjust to a stoma

Eligible studies were also required to be: (i) published in English or have a full‐text English translation; (ii) primary, peer‐reviewed studies which employed a qualitative methodology, including mixed‐methods studies in which qualitative data collection, analysis and findings were reported separately, allowing data to be extracted and synthesized independently. Studies were excluded if they had incomplete information or lacked crucial data (e.g., studies that did not discuss or provide raw data for findings related to body image).

The definition of psychological experiences used in this review was determined based on the existing literature, including empirical studies, previous reviews and theoretical frameworks which examined psychological experiences in varying contexts (Isaacs & Andipatin, 2020; Patel et al., 2025). Behavioural consequences of body image change, despite being fundamentally connected to psychological experiences, were considered secondary manifestations rather than direct reflections of internal psychological processes. As such, studies focusing on behavioural responses to body image change did not qualify as a ‘psychological experience’ within this review.

### Search strategy

A systematic search strategy was carried out by the first author to identify relevant studies across three scientific databases: MEDLINE, PsychINFO, and CINAHL, providing coverage of all key elements of the topic area including medical, psychological and nursing perspectives. Studies were searched from inception to 2nd July 2025 without date restrictions. Boolean operators (AND/OR) were used to combine keywords (searching the Title/Abstract fields) and subject headings for: (i) stoma patients, (ii) body image change and (iii) psychological experiences. Search terms were created by SL based on preliminary research into the subject area (including other systematic reviews), and with the support of a subject librarian and the third author (MB), who has clinical experience working psychologically with bodily changes.

The final keyword search terms were:

- Stoma patients: stoma OR ostomy OR colostomy OR ileostomy OR urostomy OR enterostomy OR colonic pouches OR ‘surgical stoma’
- Body image change: ((body‐image OR self‐image OR ‘body dissatisfaction’ OR ‘body satisfaction’ OR ‘appearance dissatisfaction’ OR ‘appearance satisfaction’ OR body‐perception OR ‘body image decline’ OR body‐esteem OR body‐accept* OR body‐positiv* OR body‐identit* OR self‐perception OR ‘body perception’ OR ‘body representations’) OR ((body or appearance) N4 (negative* OR dissatisf* OR critical* OR decline OR shame OR ashamed)))
- Psychological experiences: ((psycholog* OR cogniti* OR thought* OR think* OR attitude OR concept* OR schema OR self‐schema OR assumptions OR ideal* OR standard* OR judgement OR image* OR percept* OR internal* OR emot* OR feeling* OR affect OR fear OR shame) OR ((psycholog*) N4 (impact OR experience OR effect OR process OR change OR construct OR factor OR effect)))

Following this, backwards citation chaining was implemented across the articles included after screening. The full search strategy for each database can be found in Appendix A.

### Data abstraction

Data were imported into the reference management software EndNote 21. Duplicates were first removed from the database records automatically using EndNote and then manually checked by SL. One reviewer (SL) screened studies for eligibility in line with inclusion and exclusion criteria. A two‐step screening process was implemented. All identified studies were initially screened as titles and abstracts before the full texts of the remaining results were screened to determine eligibility. Studies that could not be determined as eligible from the title and abstract alone were included in the full text screening. Any discrepancies were resolved through discussion with a second reviewer in the research team. Findings related to the psychological experience of body image were extracted manually by the first researcher onto a standardized data extraction form (see Appendix B).

### Quality assessment

The widely used Joanna Briggs' Institute (JBI) Checklist for Qualitative research was employed to evaluate the methodological quality of the included studies (Hannes et al., 2010; Lockwood et al., 2015). Its focus on examining the congruency of a study's philosophical and methodological foundations enables the assessment of credibility, transferability, dependability and confirmability, to determine if studies are both well‐designed *and* meaningfully interpretative (Hannes et al., 2010). Compliance with items on the JBI checklist is indicated by ‘yes’ or ‘no’ responses. An ‘unclear,’ or ‘not applicable’ option is available for studies which do not provide sufficient information to make a definitive judgement, or for which a specific criterion is irrelevant to the study design. Response options were amended to include a ‘partial’ option which enabled consideration of studies which had met some elements of the criterion but not others.

SL and SB independently assessed the quality of each study. Scores were then cross compared, with any discrepancies being managed through discussion and comparison of rationale. The outcomes of quality assessment were not used to exclude studies as intended by the JBI checklist. Instead, the overall methodological quality of the studies was calculated using the approach of a previous review (de Smalen et al., 2021). Responses were scored (yes = 2, partial = 1, no/unclear = 0) and the percentage of compliance with items on the JBI checklist was calculated based on each study's highest possible total (to account for ‘not applicable’ items). In the JBI checklist, items 4–8 are associated with methodological bias. As such, it was also possible to calculate the risk of bias for each study by reversing the percentage compliance score for these items. This provided an additional layer of methodological insight, allowing for critical reflection on the trustworthiness of findings. Low quality/ risk of bias was represented by scores of 0–49.9%, moderate by 50–74.9% and high by 75% and above (de Smalen et al., 2021). Detailed notes were recorded of this process to maximize transparency and consistency in the quality appraisal.

### Data synthesis

Data were synthesized by SL using meta‐ethnography. Meta‐ethnography is an inductive approach for meta‐synthesis, recommended for qualitative data which explore subjective and nuanced phenomena, like that of psychological experiences of body image change. This approach supports the re‐interpretation of primary research while preserving the emotional and contextual nuance of participants' narratives, to identify broader, contextual patterns and complexities in participants' overall accounts (Noblit & Hare, 1988). From this, new interpretations known as ‘third order constructs’ can be depicted which aim to reflect a deeper understanding of the phenomenon in question (Noblit & Hare, 1988). As described by Britten et al. (2002), meta‐ethnography ‘cannot be reduced to a set of mechanistic tasks’ and is often a highly iterative and non‐linear process which values conceptual contribution over methodological perfection. As such, guidance on how to complete a meta‐ethnography can vary. Meta‐ethnography in the current review was carried out following the stages outlined by Sattar et al. (2021), as detailed below.

In each study, the key themes and metaphors regarding the psychological experience of body image were identified, cross‐compared and grouped (according to similarity) to determine the initial overarching ‘constructs’ across the data. A construct is defined as ‘having some analytical or conceptual power, unlike more descriptive themes’ (Britten et al., 2002). Initial constructs were displayed in a table along with the supporting primary data, transcribed verbatim to preserve meaning and reduce researcher bias. All key themes and metaphors were encompassed by the concepts, making it an iterative process where interpretations were continually refined in light of the data. Studies were ordered chronologically during this stage to support the exploration of concept development over time. From this, constructs and the supporting data were compared across and within studies, and alongside contextual, methodological and demographic information, to determine the conceptually similar or opposing ideas that ran across studies. In a meta‐ethnography, this is known as ‘translation’. These emerging ideas were then analysed using: (i) reciprocal translation, (ii) refutational synthesis and (iii) line of argument synthesis, as originally outlined by Noblit and Hare (1988). To express the synthesis, the eMERGe guidance was followed (France et al., 2019). The outcome of this process is presented in Supplementary Materials B and C.

### Confidence in the review findings

After completing the synthesis, SL assessed confidence in each third‐order construct using the Confidence in the Evidence from Reviews of Qualitative Research (GRADE‐CERQual) approach (Lewin et al., 2018). For each construct, judgements considered four components: methodological limitations, coherence, adequacy of the contributing data and relevance to the review question. SL made the initial component and overall confidence judgements, which MB checked against the extracted data, quality appraisal and synthesis. Differences were resolved through discussion between SL and MB.

### Reflexivity

Reflexivity is an essential component of qualitative research which encourages researchers to critically examine how their role, background and experiences might shape the research process. Olmos‐Vega et al. (2023) stated that reflexivity can support the interpretive trajectory by deepening the understanding of how meaning is created.

In the current review, the research team brought complementary perspectives to this process. SL is a Trainee Clinical Psychologist with clinical, theoretical and research experience related to body image. This supported sensitivity to appearance‐related distress but also required attention to the possibility of interpreting findings through existing body‐image models. SL therefore kept a reflexive diary throughout the synthesis to record personal responses to the data, analytic decisions and points of uncertainty. MB is a Clinical Psychologist with experience in psycho‐oncology and mechanism‐based psychological intervention development. This brought clinical and translational relevance to the synthesis, while also prompting reflection on whether interpretations were being shaped too strongly by a cancer‐care or intervention‐development lens. SB brought specialist expertise in qualitative methodology and supported attention to the distinction between participant accounts, primary authors' interpretations and the review team's third‐order constructs.

Reflexive discussion took place through supervision and team review, with attention to grounding interpretations in the extracted data, considering alternative explanations and identifying refutational cases. The author team did not include a person with lived experience of living with a stoma, which made it particularly important to remain close to participants' accounts and to avoid assuming experiential meanings not supported by the primary studies.

## RESULTS

### Systematic search results

A total of 843 records were identified: 790 through database searches and 53 through backwards citation chaining. After removing 221 duplicates, 569 database records remained for title and abstract screening. Of these, a further 504 were excluded. The remaining 65 database records and the 53 records from citation chaining (118 in total) were sought for full‐text retrieval; however, 25 were inaccessible online. Attempts were made to contact authors, but no responses were received within the allocated timeframe, and these studies were excluded. After full‐text screening, a further 75 studies were excluded. This process is summarized in Figure 1.

**FIGURE 1:**
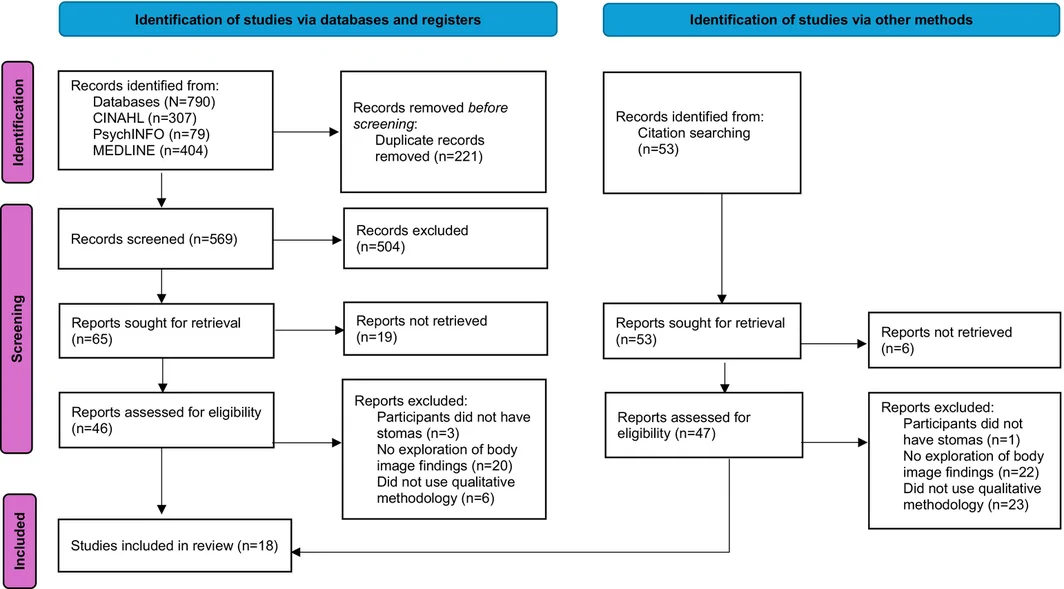
PRISMA flow diagram.

### Study characteristics

Table 1 outlines the characteristics of the 18 studies included in this review. The total sample size was 602. Gender was reported for 589 participants across 17 studies: 324 (55%) were reported as male and 265 (45%) as female. Gender was not reported for 13 participants across 1 study. Participants' diagnoses were varied, including: cancer (colorectal, rectal, colon, bladder, gynaecological), fibrosis, colon necrosis, inflammatory bowel disease, fistula, ulcerative colitis, perforated appendix, diverticulitis, Crohn's disease, endometriosis, familial polyposis and physical trauma. Participants had a range of temporary (48%) or permanent (52%) ileostomies, colostomies and urostomies.

**TABLE 1:** Study characteristics.

Study	Author/s (year); country	Sample characteristics	Age (mean in years)	Time since surgery	Methodological approach theoretical underpinnings; study design; data analysis	Key body image findings
1	Kandemir and Oskay (2017); Turkey	Gender: Male (*n* = 10) Diagnosis: Bladder cancer (*n* = 10) Type of stoma: Urostomy (*n* = 10) Stoma Permanence: NS Total (*N* = 10)	59.9	≥6 months	Phenomenology; NS; interviews (US); content analysis	Participants experienced a loss of bodily integrity and heightened embarrassment following urostomy surgery.
2	Petersén and Carlsson (2021); Sweden	Gender: Male (*n* = 9) Female (*n* = 12) Diagnosis: CRC (*n* = 14) IBD (*n* = 5) Gynaecological cancer (*n* = 1) Fistula (*n* = 1) Type of stoma: Colostomy (*n* = 13) Ileostomy (*n* = 8) Stoma Permanence: NS Total (*N* = 21)	65	1–3 years	NS; semi‐structured focus groups; content analysis	Participants felt anxious about the visibility of their stoma and feared negative evaluation from others. Many wore loose fitted clothing to control this. Some began to buy clothes they liked and felt less ashamed of their appearance.
3	Reisdorfer et al. (2019); Brazil	Gender: NS Diagnosis: Cancer (US, *n* = 9) NS (*n* = 4) Type of stoma: Colostomy (*n* = 9) NS (*n* = 4) Stoma Permanence: Permanent (*n* = 3) Temporary (*n* = 10) Total (*N* = 13)	60.5	≥3 months	NS; NS; semi‐structured interviews; thematic analysis	Participants felt unattractive. Some experienced significant distress when thinking about spending their life with a stoma. Women felt more ashamed of their body than men.
4	Beitz (1999); USA	Gender: Male (*n* = 4) Female (*n* = 6) Diagnosis: UC (*n* = 1) UC with RC (*n* = 9) Type of stoma: NS Stoma Permanence: NS Total (*N* = 10)	37.7 at time of surgery	2.6 years (mean)	Phenomenology; NS; interviews (US); NS	Participants felt ‘different’ following stoma surgery. The stoma was often referred to in the third person. This change was highly distressing.
5	Vural et al. (2016); Turkey	Gender: Male (*n* = 7) Female (*n* = 7) Diagnosis: CRC (*n* = 10) IBD (*n* = 4) Type of stoma: Colostomy (*n* = 7) Ileostomy (*n* = 6) Urostomy (*n* = 1) Stoma Permanence: Permanent (*n* = 6) Temporary (*n* = 6) NS (*n* = 1) Total (*N* = 14)	45.3	2–12+ months	Phenomenology; NS; semi‐structured interviews; content analysis	Some participant indicated that their feelings of attractiveness were not changed, but others described feeling intense dissatisfaction.
6	Sammut et al. (2017); Malta	Gender: Male (*n* = 4) Female (*n* = 6) Diagnosis: UC (*n* = 10) Type of stoma: Ileostomy (*n* = 10) Stoma Permanence: NS Total (*N* = 10)	NS	5.4 years (mean)	Phenomenology; NS; semi‐structured interviews; IPA	Participants' mental representation of their body significantly changed. Many struggled with the visibility of stoma surgery and perceived a deterrence from body image standards. Some avoided mirrors due to shame. Body image distress particularly impacted young people.
7	Cardoso et al. (2015); Brazil	Gender: Male (*n* = 6) Female (*n* = 4) Diagnosis: NS Type of stoma: NS Stoma Permanence: NS Total (*N* = 10)	NS	≥1 year	NS; NS; interviews (US); content analysis	Stoma formation led to feelings of ugliness. Participants attempted to hide this from themselves and those around them. This change was perceived to compromise participants' ability to satisfy social expectations of the body. For many, this was interlinked with their identity as a woman.
8	González et al. (2016); Sweden	Gender: Male (*n* = 183) Female (*n* = 137) Diagnosis: RC (*n* = 320) Type of stoma: NS Stoma Permanence: NS Total (*N* = 320)	Mean NS, median = 65	≥3 years	Mixed methods; NS; open‐ended questionnaire; content analysis	Participants were ashamed of their altered body and described themselves as ugly. Female participants felt that the stoma had reduced their femininity. Participants who experienced a feeling of health and wellbeing following surgery felt able to accept the change.
9	Tomasi et al. (2022); Brazil	Gender: Male (*n* = 35) Female (*n* = 42) Diagnosis: NS Type of stoma: Colostomy (*n* = 60) Ileostomy (*n* = 17) Stoma Permanence: NS Total (*N* = 77)	NS	NS	NS; NS; semi‐structured interviews; content analysis	Many felt anxious and ‘different’ to their peers. They thought others would negatively judge their appearance.
10	Mathieu et al. (2025); Switzerland	Gender: Male (*n* = 5) Female (*n* = 4) Diagnosis: CRC (*n* = 9) Type of stoma: Colostomy (*n* = 9) Stoma Permanence: Permanent (*n* = 9) Total (*N* = 9)	73.1	16.4 years (mean)	NS; critical realism; semi‐structured interviews; thematic analysis	Many felt shame when looking at their body but coped with this by personifying the stoma.
11	Persson and Hellström (2002); Sweden	Gender: Male (*n* = 5) Female (*n* = 4) Diagnosis: RC (*n* = 2) UC (*n* = 3) BC (*n* = 2) Diverticulitis (*n* = 1) Rectovaginal fistula (*n* = 1) Type of stoma: Colostomy (*n* = 5) Ileostomy (*n* = 2) Urostomy (*n* = 2) Stoma Permanence: NS Total (*N* = 9)	53.3	6–12 weeks	Phenomenology; NS; unstructured interviews; NS	Participants felt alienated from their body following surgery. Many were able to adjust to this change when returning to their home environment but continued to feel like their attractiveness had decreased.
12	Thorpe et al. (2016); UK	Gender: Male (*n* = 6) Female (*n* = 6) Diagnosis: RC (*n* = 5) UC (*n* = 2) Diverticulitis (*n* = 4) Crohn's disease (*n* = 1) Type of stoma: Colostomy (*n* = 9) Ileostomy (*n* = 3) Stoma Permanence: Permanent (*n* = 6) Temporary (*n* = 6) Total (*N* = 12)	NS, range = 30's‐70's	3–15 months	Phenomenology; NS; unstructured interviews; NS	The significance of bodily changes fluctuated across time. Many felt initial shock and horror towards the stoma. Some felt that familiarity facilitated acceptance, but continued to experience concern about the appearance of the stoma under clothes.
13	Savard and Woodgate (2009); Canada	Gender: Male (*n* = 1) Female (*n* = 5) Diagnosis UC (*n* = 6) Type of stoma: NS Stoma Permanence: Temporary (*n* = 6) Total (*N* = 6)	NS, range 19–24	1–8 years	Phenomenology; NS; semi structured interviews; NS	Participants felt embarrassed about their body. They initially wanted to hide their stoma but learned to accept their appearance by returning to their normal daily activities.
14	Smith et al. (2017); UK	Gender: Male (*n* = 10) Female (*n* = 11) Diagnosis: UC (*n* = 11) BC (*n* = 3) Crohn's disease (*n* = 5) NS (*n* = 2) Type of stoma: Ileostomy (*n* = 21) Stoma Permanence: NS Total (*N* = 21)	46.8	2–3 years	Hermeneutic phenomenology; NS; structured interviews; IPA	Participants experienced their bodies as unfamiliar leading to a sense of being ‘attacked’ by the stoma. Some began to feel desensitized. This was often supported by participants disidentifying with their changed body.
15	Hueso‐Montoro et al. (2016); Spain	Gender: Male (*n* = 12) Female (*n* = 9) Diagnosis: Cancer (US, *n* = 14) IBD (*n* = 6) Familial polyposis (*n* = 1) Type of stoma: Colostomy (*n* = 11) Ileostomy (*n* = 10) Stoma Permanence: Permanent (*n* = 13) Temporary (*n* = 8) Total (*N* = 21)	NS, range 20–75	NS	Phenomenology; NS; semi structured interviews; NS	Some rejected their appearance following stoma surgery, whereas some were able to integrate this into their bodily identity by personifying the stoma or using humour. Many were not able to experience acceptance which led to ongoing concerns about how their stoma would influence others' judgement.
16	Marques et al. (2018); Brazil	Gender: Male (*n* = 6) Female (*n* = 4) Diagnosis: CRC (*n* = 2) UC (*n* = 1) White‐hole puncture (*n* = 1) Trauma (*n* = 1) Intestinal tumour (*n* = 1) Diverticulitis (*n* = 1) Crohn's disease (*n* = 1) Endometriosis (*n* = 1) NS (*n* = 1) Type of stoma: Colostomy (*n* = 7) Ileostomy (*n* = 3) Stoma Permanence: NS Total (*N* = 10)	49	6 months‐12 years	Phenomenology; NS; interviews (US); NS	Participants felt that their altered body was strange. Some experienced denial and perceived the stoma as separate from themselves.
17	Villa et al. (2018); Italy	Gender: Male (*n* = 9) Female (*n* = 2) Diagnosis: NS Type of stoma: Urostomy (*n* = 11) Stoma Permanence: NS Total (*N* = 11)	69	NS	Phenomenology; NS; semi‐structured interviews; IPA	Some stated that the urostomy did not impact their body image, but others felt disturbed when looking at the stoma.
18	Palomero‐Rubio et al. (2018); Spain	Gender: Male (*n* = 12) Female (*n* = 6) Diagnosis: Colon cancer (*n* = 18) Type of stoma: Colostomy (*n* = 18) Stoma Permanence: NS Total (*N* = 18)	NS	NS	Phenomenology; NS; interviews (US); content analysis	Participants experienced a significant change in body image following surgery which was often related to their perception that their body differs from societal beauty standards. Participants who accepted their body described seeing it as something independent to the stoma.

Abbreviations: BC, bladder cancer; CRC, colorectal cancer; IBD, inflammatory bowel disease; IPA, interpretative phenomenological analysis; NS, not stated; RC, rectal cancer; UC, ulcerative colitis; UK, United Kingdom; US, unspecified; USA, United States of America.

### Quality appraisal

Table 2 outlines the results of the quality appraisal using the JBI checklist. Methodological quality ranged from 50 to 85% (moderate–high) and risk of bias ranged from 10 to 70% (low–moderate). The mean across all studies was 65.8% for methodological quality (moderate) and 46.7% (low) for risk of bias. No studies were at high risk of bias under the stated thresholds. All studies appeared to have partial or full congruence between the methodological approach and methods used to collect data (Q2–Q3); however, only one study fully and two studies partly stated a philosophical position (Q1). Just two studies adequately acknowledged the researcher's background (Q6), while three sufficiently addressed reflexivity (Q7). Twelve studies stated that they had adopted a phenomenological approach, but five of these did not explore single reported or divergent experiences (Q4). Similarly, five studies did not fully represent participants' voices in their results (Q8). With regards to findings, 72.2% demonstrated full congruence between their methodology and interpretation of the results (Q5) and 72.2% between their interpretation of the results and the conclusions drawn (Q10). All studies demonstrated that ethical approval processes had been followed (Q9).

**TABLE 2:** Quality appraisal of each study using the Joanna Briggs' Institute Qualitative Checklist.

Study	JBI checklist item										Compliance (%)	Risk of bias (%)
	1	2	3	4	5	6	7	8	9	10		
1	Unclear	Yes	Yes	Yes	Yes	No	No	Yes	Yes	Yes	70% (moderate)	40% (low)
2	Unclear	Yes	Yes	Unclear	Yes	Partial	Yes	Yes	Yes	Yes	75% (high)	30% (low)
3	Unclear	Yes	Yes	Unclear	Yes	No	No	Yes	Yes	Yes	60% (moderate)	60% (moderate)
4	Unclear	Yes	Yes	Yes	Yes	Yes	No	Partial	Yes	Yes	75% (high)	30% (low)
5	Unclear	Yes	Yes	Partial	Yes	No	No	Partial	Yes	Partial	55% (moderate)	60% (moderate)
6	Unclear	Yes	Yes	Yes	Yes	No	No	Yes	Yes	Partial	65% (moderate)	40% (low)
7	Unclear	Yes	Yes	Partial	Partial	No	Yes	Yes	Yes	Yes	70% (moderate)	40% (low)
8	Unclear	Yes	Partial	Partial	Yes	No	No	Partial	Yes	Yes	55% (moderate)	60% (moderate)
9	Partial	Yes	Yes	Unclear	Yes	No	No	Partial	Yes	Yes	60% (moderate)	70% (moderate)
10	Partial	Yes	Yes	Unclear	Unclear	No	Yes	Yes	Yes	Yes	65% (moderate)	60% (moderate)
11	Unclear	Yes	Yes	Yes	Yes	No	No	Yes	Yes	Partial	65% (moderate)	40% (low)
12	Unclear	Yes	Yes	Yes	Yes	Yes	Partial	Yes	Yes	Yes	85% (high)	10% (low)
13	Unclear	Yes	Yes	Partial	Yes	No	No	Partial	Yes	Unclear	50% (moderate)	60% (moderate)
14	Unclear	Yes	Yes	Yes	Unclear	No	Partial	Yes	Yes	Yes	65% (moderate)	50% (moderate)
15	Unclear	Yes	Yes	Yes	Partial	No	No	Yes	Yes	Yes	65% (moderate)	50% (moderate)
16	Yes	Yes	Yes	Yes	Yes	No	No	Yes	Yes	Yes	80% (high)	40% (low)
17	Unclear	Yes	Yes	Yes	Yes	No	No	Yes	Yes	Yes	70% (moderate)	40% (low)
18	No	Yes	Yes	Partial	Partial	No	Unclear	Yes	Yes	Partial	55% (moderate)	60% (moderate)

### Synthesis

Three third‐order constructs and five sub‐themes were found across two time phases: immediately after surgery and living with the stoma (see Figure 2). First, immediately after stoma surgery, ‘*All‐Consuming Shock*’ captured participants' profound distress and sense of bodily unfamiliarity. As participants transitioned to living with the stoma, *Visible* ‘*Otherness*’ described how this disruption became embedded within everyday life and ‘*Fluctuating Acceptance*’ illustrated that adaptation was neither linear nor complete; instead, participants continually negotiated acceptance of their changed body through psychological coping strategies and external sources of validation.

**FIGURE 2:**
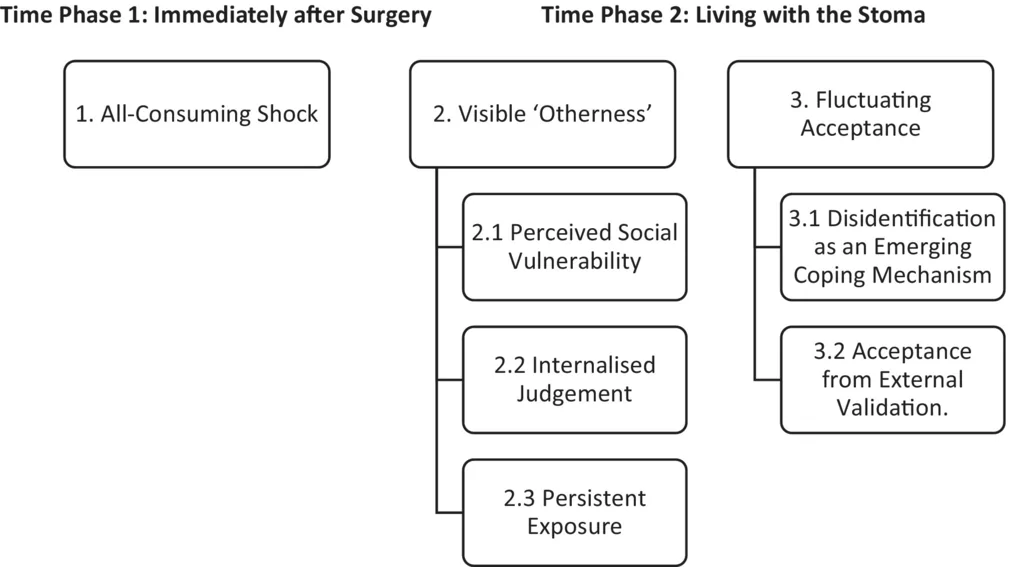
The three third‐order constructs and associated sub‐themes across the two time phases of participants' experiences.

Themes were supported by between six and 18 studies. Findings are summarized in Table 3, and the initial evaluation for the GRADE‐CERQual can be found in Supplementary Materials D.

**TABLE 3:** Themes and sub‐themes in each study.

Time phase	Third‐order construct *sub‐theme*	Study number																		GRADE‐CERQual confidence
		1	2	3	4	5	6	7	8	9	10	11	12	13	14	15	16	17	18	
Immediately after surgery	All‐consuming shock			*						*		*	*			*	*			Moderate
Living with the stoma	Visible ‘Otherness’																			Moderate
	*Perceived social vulnerability*	*	*	*	*		*	*	*	*	*	*	*	*	*	*	*		*	
	*Internalized judgement*		*	*	*	*	*		*	*		*	*		*			*		
	*Persistent exposure*		*				*	*			*		*	*	*	*	*	*		
	Fluctuating acceptance																			Moderate
	*Disidentification as an emerging coping mechanism*				*						*		*		*	*	*		*	
	*Acceptance from External validation*	*				*		*	*			*		*		*	*	*		

*Note*: *indicates that the study is included in the third‐order construct or sub‐theme.

### Time phase 1: Immediately after surgery

#### All‐consuming shock

Participants in six studies (3, 9, 11, 12, 15, 16) experienced significant ‘shock’ and ‘horror’ towards their body post‐surgery. Confidence in this finding was rated as moderate, reflecting methodological limitations within the contributing studies and moderate concerns regarding the adequacy of the available data. Many were ‘unable to look’ at their altered body and described it as ‘unfamiliar’, rejecting the idea of this change being permanent. This often led to extreme distress:

I hope the doctor does not tell me it is forever. Otherwise, I will commit suicide. (3)

… an invalid for the rest of my life. (12)

It is at this time where the stoma was appraised as a ‘threat’ to the person's body (e.g., ‘alien,’ ‘unnatural’). Often, participants could not integrate the change into the mental representation of their appearance to experience bodily unity:

It caused me strangeness to come back from surgery and see. (16)

Together, this suggests that participants' early post‐surgery reactions to their altered body represent not only distress, but a profound rupture in bodily integrity, where the stoma becomes a symbol of strangeness that patients struggle to reconcile with their pre‐surgery appearance.

### Time phase 2: Living with the stoma

#### Visible ‘otherness’

Participants in all 18 studies discussed a shift in their mental representation of their body, encompassing participants' internal critic *and* their perceptions of how the body is viewed by others. Reciprocal translation revealed that ‘visible otherness’ was often centred around the visibility of the stoma and its disruption of body ‘normality’. Confidence in this construct was moderate. Although the finding was coherent, relevant and supported by rich data across all 18 studies, methodological concerns, particularly limited reflexivity and epistemological transparency, prevented a higher rating. This is captured within three sub‐themes as detailed below.

##### Perceived social vulnerability

Participants from 16 studies (1–4, 6–16, 18) described perceiving their altered body as ‘different’ from others without stomas, and perceived their appearance as a visible deviation from perceived appearance norms. This left participants feeling vulnerable to social judgement, and they often evaluated their body image through imagining ‘disgust’ of others:

I don't think that's nice for other people to see such a thing… you wouldn't feel comfortable walking around showing your tummy off. (12)

… I used to think that people were looking at it… I can say that at that time I was really depressed. (6)

Female participants discussed this process more explicitly, describing how the presence of a stoma intersected with gendered appearance expectations. For many women, stoma surgery destabilized their sense of femininity within their body:

… it is horrible to see it hanging there. I find the scar ugly, I do not like looking in the mirror. When I looked in the mirror, it looked like I was not going back to being a woman. (7)

The same was seen for age‐related concerns, with a young female describing:

… because of my age, you have just started life… and you have a scar… I do not feel comfortable. (6)

Overall, body image change for participants was characterized by a negotiation of social adequacy, where the social emphasis placed on appearance becomes the driver of how visible difference is experienced or resisted.

##### Internalized judgement

Participants in 11 studies (2–6, 8, 9, 11, 12, 14, 17) described an internal dialogue marked by self‐criticism and perceived inadequacy when discussing bodily changes. Across these studies, participants related their appearance to the views of others. While closely linked to the social gaze, this reflected a more internal process where societal norms and others' imagined evaluations were internalized and became an implicit part of participants' own self‐appraisals:

… I was prejudiced against myself … I thought it was a strange body. (9)

Many described their altered body as ‘less attractive,’ with some using terms like ‘ugly’ and ‘mutilated,’ reflecting how body image changes following stoma surgery can be profound for some:

… well, you do feel mutilated really, you look down and here is this horrible thing. (12)

Overall, internalized standards of attractiveness shaped participants' appraisals of their body, fuelling an ongoing cycle of self‐surveillance and self‐criticism.

##### Persistent exposure

Across 10 studies (2, 6, 7, 10, 12–17), participants articulated feeling shame following stoma surgery. Although accounts differed in the emotion used to describe this (‘shame’, ‘embarrassment’), this was related to how the physical protrusion of the stoma and its appearance under clothes were experienced as unavoidable breaches of bodily privacy. The altered body was perceived as ‘more open and less private’ and ‘hard to conceal’, providing useful metaphors to elucidate how the stoma's visibility can contribute towards an experience of social and intrapersonal exposure:

… all I was worried about was how I can wear things so nobody else would see it. (12)

…when I look at myself in the mirror… [I feel] a bit of discomfort from the aesthetic point of view… it's that what psychologically disturbs me. (17)

In contrast, young women in one study were comfortable wearing tight clothes, but connected this to the security of keeping the stoma bag in place (2).

These accounts portray bodily shame as arising from a perceived blurring of the boundary between the private and public bodily self, transforming the body into a site of continued exposure and vulnerability.

#### Fluctuating acceptance

Across 14 studies (1, 4, 5, 7, 8, 10–18), appraisals of their altered bodies fluctuated across contexts. Because primary studies rarely differentiated findings by cultural or clinical subgroups, the synthesis could not determine whether this fluctuation varied by culture or illness factors. Instead, the availability of internal and external coping resources appeared central to moments of acceptance. Confidence in this construct was moderate because, although the finding was coherent and relevant, some contributing studies had methodological limitations and provided limited richness and quantity of data.

##### Disidentification as an emerging coping mechanism

Participants in seven studies (4, 10, 12, 14–16, 18) described stoma surgery as having led to a fragmentation in their bodily identity and talked about their stoma as separate from themselves. While many authors described this as indicative of bodily distress, it often appeared within participants' quotations as a protective mechanism that participants used to embrace their altered body:

It's assuming that it's there and that's it … like a cell phone in your pocket or a pack of gum. (15)

Over time, participants' disidentification with the stoma enabled a cognitive dissonance where the dissatisfaction with the appearance of the stoma became simultaneous with their acceptance of bodily change:

… nothing wrong about me, no! It's only the bag. (16)

Some participants incorporated humour and playfulness into this process, describing the stoma as ‘amusing.’ While many participants were able to utilize psychological distancing, one refutational case experienced her altered body as deeply distressing and described the stoma as an ‘alien’ which attacks her in her dreams:

… the stoma breaks out [of the body]. And the scar forms… they're horrible, horrible dreams. (14)

However, there was a suggestion from the authors that this was related to the chronic shame and fear felt by this individual regarding the ‘unnatural’ and ‘imperfect’ body that she perceived.

The tendency to disidentify with the stoma reflects a complex psychological strategy through which individuals manage the disruption to their bodily identity. Though for some, especially those burdened by shame or internalized ideals of bodily perfection, this separation may amplify distress rather than alleviate it.

##### Acceptance from external validation

Participants in nine studies (1, 5, 7, 8, 11, 13, 15, 16, 17) discussed developing acceptance of their altered body when paired with external sources of validation. Specifically, affirmation from family members and intimate partners, engagement with beauty treatments, or success in occupation appeared to reduce the impact of appearance‐related concerns:

… as I arrive in front of the mirror and look at myself, despite having a bag, I know that I am happy because I am a very successful person. (7)

I have accepted my body, my husband has been so helpful all the time. (8)

Environments where bodily appearance was acknowledged without judgement, or where participants could succeed in their usual activities, offered a sense of normality which helped participants to view the body as a symbol of ‘strength and survival’. Interestingly, this also appeared to occur simultaneously with participants' perception of bodily ‘difference’ and perceived appearance norms:

… here, my wife and daughter provided the most contribution, but not wearing trousers is uncomfortable. (1)

Conversely, when family support was not available, one participant was not able to accept their altered body. The author interpreted this as a continued sense of ‘strangeness’ in their body:

… my family said… ‘it's very disgusting!’… It is difficult to find such a new person with the courage to exhibit. (16)

This sub‐theme presents bodily acceptance as deeply relational, where others' validation can allow individuals to reframe their altered bodies as resilient, and provide distance from feelings of shame and inadequacy.

### Line of argument synthesis

The initial shock experienced towards the body following stoma surgery represented a profound threat to bodily integrity and sense of self. This disruption prompted participants to engage in a process of psychological reconstruction, appraising their altered appearance against cultural ideals and perceived social judgments. Many concluded their body was now ‘different,’ leading to heightened self‐consciousness and shame. In seeking to reduce this distress and avoid judgement from others, participants attempted to conceal their bodies and hide their stoma. However, the stoma's visibility made concealment difficult, leaving many with a persistent sense of exposure and vulnerability. Over time, these factors became internalized and entangled with participants' self‐appraisals, reinforcing cycles of self‐surveillance and self‐criticism. To manage this ongoing tension, participants sought moments of acceptance through internal strategies such as psychological distancing and disidentification, and through external sources of validation from supportive relationships or valued social activities. Drawing on these strategies varied across people and over time, causing body image satisfaction to fluctuate, oscillating between personal peace and social distress.

## DISCUSSION

To the authors' knowledge, this is the first review to examine the psychological experience of body image in stoma patients. Eighteen studies were synthesized using meta‐ethnography, highlighting three main themes across two time phases: (1) all‐consuming shock, (2) visible ‘otherness' and (3) fluctuating acceptance.

The influence of socially constructed body image standards on participants' psychological experiences was the most prominent theme and was evident across all included studies. Consistent with research in related fields, body image was experienced as a socially mediated interface in which appearance standards shaped participants' subsequent thoughts and emotions, particularly regarding perceived attractiveness (Bergstrom & Neighbors, 2006). Although dominant body image theories suggest that some appearance concerns (e.g., shape and weight) are influenced by specific cultural ideals, the psychological experience of body image change in stoma patients was similar across all settings in this review. It is possible that the visibility of the stoma may signify a form of bodily difference which evokes similar psychological responses across different social contexts, highlighting shared beauty standards (e.g., ideals of bodily wholeness, symmetry and physical integrity) that are rooted less in the pursuit of cultural ideals, and more in the symbolic meaning of a medicalised body.

Participants' sense of dissatisfaction with their altered body also emerged despite differences in diagnoses and other medical factors across the included studies. This is in line with previous reviews which demonstrate that body image distress is a common psychological consequence of stoma formation, regardless of indication (Ayaz‐Alkaya, 2019). This review adds that some individuals were able to experience acceptance of their changed body when utilizing psychological distancing or external sources of validation. Such strategies appear to reduce participants' focus on the stoma and enhance their sense of self‐worth, providing one possible explanation for why body image distress varied between individuals despite similar bodily changes. Altschuler et al. (2009) found that husbands' support post‐surgery increased levels of psychosocial adjustment and flexibility, which would support this argument. However, a striking contrast emerged between this and the persistent negative influence of social variables on bodily appraisals. Perhaps this form of acceptance functions less to restore feelings of attractiveness and more as a coping mechanism which provides temporary relief from emotional distress, enabling individuals to maintain psychological stability and functioning in the face of irreversible bodily change.

In contrast, it could be argued that some strategies used to facilitate acceptance instead perpetuate the underlying body‐related distress that persists over time. For example, the use of disidentifying language when describing the stoma might inhibit the integration of an individual's changed body into their self‐concept. This interpretation aligns with traditional psychological models of body image, which propose that avoidance and other safety behaviours may reduce distress in the short term while maintaining body image difficulties over time (Cash, 2012; White, 2000). It is also consistent with the present synthesis, in which acceptance was not experienced as a stable or enduring state but instead fluctuated over time and place, suggesting that these strategies may alleviate distress without fully resolving it. While language adaptations and engagement with valued activities could be highlighted as important targets for psychological intervention in this area, it is important to consider *how* these strategies are used, ensuring they promote long‐term, meaningful adaptation to life with a stoma, rather than temporary coping with it.

Further to the above, because participants' negative self‐appraisals first appeared alongside the initial shock of viewing their altered body, it is possible that unpreparedness for this change may exacerbate awareness of social ideals and amplify the internalization of stigma. Person et al. (2012) found that stoma site marking can reduce levels of bodily embarrassment post‐surgery, which would support this finding. However, several factors could confound this interpretation. Participants' pre‐surgical experiences of body image were not included in the findings of any of the included studies. Similarly, some participants' description of the altered body as a symbol of vitality may point towards the influence of illness severity *before* the stoma, a factor which was also not considered. Because many studies were only partially focused on body image, the interpretive depth of authors' original findings was limited at times. This may have subsequently limited the depth of the current findings, and it is possible that additional meaning would be available should the data have explored body image more broadly. Future research should focus on distinguishing how factors like previous body image distress, social support and illness severity impact the psychological experience of body image change for these patients, to clarify individual variability and identify those most at risk of poor psychological outcomes.

## LIMITATIONS

A breadth of diagnoses, countries, participant characteristics and clinical contexts were represented in the included studies, enabling the identification of recurring patterns and similarities in the psychological experience of body image change across these groups, consistent with the aims of meta‐ethnography. The review included studies from Brazil and Turkey, alongside European and North American settings, providing some opportunity to consider if these experiences were shared across different cultural settings too. However, primary study samples were often heterogeneous in terms of more specific subgroups like age, ethnicity, gender and stoma status, and authors rarely differentiated this within their findings, precluding robust comparisons in this way. The transferability of the constructs across these groups therefore remains uncertain, and future studies should aim to report and analyse contextual differences more explicitly.

Additionally, many studies lacked epistemological transparency, making it difficult to locate authors' interpretations within a clear theoretical or ontological framework. This was reflected in moderate CERQual concerns regarding methodological limitations across several findings, and constrained the ability to explore how researchers' assumptions shaped their analysis of body image change. While most maintained coherent links between research aims, data collection and analysis, quality appraisal found that participants' voices were also not well represented across several of the included studies. However, these limitations reflect broader methodological tension within qualitative evidence synthesis. Although frameworks such as the JBI Checklist and GRADE‐CERQual increase the transparency and rigour of qualitative systematic reviews, they require the standardization of research that is inherently interpretive and context‐dependent. Consequently, judgements about methodological quality, risk of bias and confidence in the evidence should be understood as indicators of the trustworthiness of the synthesis, rather than objective measures of the validity or strength of participants' lived experiences. Nevertheless, transparent reporting of philosophical positioning and reflexive engagement remains essential to support consideration of how authors' interpretations were shaped, or to evaluate findings within their intended interpretative context. Future research in this area should therefore prioritize greater transparency in methodological approaches and reflexivity.

Lastly, the selection, abstraction and synthesis of studies was conducted by one author. While efforts were made to maintain reflexivity, single author qualitative research has been criticized for its limited triangulation and potentially introducing bias to the research process (Denzin & Lincoln, 2011). Taken together, this may limit the dependability of the current findings, and future research would benefit from a collaborative synthesis which prioritizes a broader spectrum of cultural frameworks worldwide.

### Clinical implications

The mechanisms identified in this review offer insights into how body image distress develops and is maintained over time and could be used to inform psychological support for this population. Several contemporary approaches to body image intervention focus on promoting self‐compassion, body functionality and self‐acceptance to develop a more flexible and accepting relationship with their bodies despite ongoing bodily differences, rather than attempting to change appearance‐related evaluations themself. These approaches may be especially appropriate given that distress in the current review appeared to be maintained through shame, self‐criticism and perceived social evaluation. Similar interventions have shown promise in populations experiencing appearance‐related changes following breast cancer treatment (e.g., Lewis‐Smith et al., 2018), suggesting that adapting these approaches to address the unique challenges associated with stoma formation may represent a valuable direction for future intervention development.

Some psychological responses were more or less likely to occur at different stages post‐stoma, and depending on the availability of external sources of support. This signifies the importance of offering interventions which are tailored to each individuals' recovery journey and personal/ social circumstances. For example, the initial shock experienced by participants suggests that more could be done to prepare patients for bodily change. Preoperative interventions should aim to provide compassionate education which equips patients with ways to think about and cope with bodily change *before* surgery is undertaken. Educational programmes aimed at supporting families and caregivers could reduce the impact of social stigma on body shame and support positive psychosocial outcomes. Individuals who experience stigma and rejection of the stoma are shown to be less likely to engage with stoma‐related self‐care (e.g., Zhang & Li, 2025), further highlighting the importance of addressing body image change to support long‐term adaptation and recovery outcomes in turn. Psychological support at all stages should not only address internalized judgements of the body, but also the psychologically complex reality of making sense of and coping with a visible and permanent marker of bodily change.

## CONCLUSIONS

This review moves beyond describing distress in stoma patients to explain how it develops, is maintained, and managed over time. Stoma formation can create an immediate rupture in bodily integrity and identity, reinforced by ongoing self‐scrutiny, imagined judgement from others and a persistent sense of exposure. These processes embed shame and self‐criticism into daily life and cannot be disentangled from broader social and relational influences. Patients actively attempt to protect themselves through psychological distancing from the stoma and through validation from partners, family or valued roles. Acceptance is therefore not a fixed outcome but a fluctuating state that depends on both personal coping and the social environment.

These findings suggest that support for stoma patients should begin before surgery and continue through recovery, addressing identity disruption, anticipated stigma and body‐related shame rather than only treating anxiety or depression after the fact. The mechanisms identified here (threat to bodily integrity, perceived social vulnerability, shame and self‐scrutiny) offer concrete targets for tailored intervention and for identifying people at risk of long‐term body image distress. Future research should test these mechanisms longitudinally and across diverse cultural and clinical contexts to inform preventive and mechanism‐based psychological interventions.

## AUTHOR CONTRIBUTIONS

**Siân Levell:** Writing – original draft; writing – review and editing; methodology; conceptualization; formal analysis; data curation. **Sophie Buckley:** Conceptualization; writing – review and editing; methodology; supervision. **Michael Baliousis:** Conceptualization; writing – review and editing; methodology; supervision.

## FUNDING INFORMATION

The authors have nothing to report.

## CONFLICT OF INTEREST STATEMENT

The authors declare no conflicts of interest.

## ETHICAL STATEMENT

The authors have nothing to report.

## TRIAL REGISTRATION

This review was registered prospectively on PROSPERO (registration number: CRD420251087477, protocol available at: https://www.crd.york.ac.uk/PROSPEROFILES/8908abe90437d4d9c750ec5adc0b1f0f.pdf).

## Supporting information


**Data S1:** Supporting Information.

## Data Availability

All relevant data are available within the manuscript and its supporting online Supporting Information files.

## APPENDIX A Search Strategy

**Table:**

	Stoma patients	Body image change	Psychological experience
Medline (EbscoHost)	(MH ‘Surgical Stomas’) OR (MH ‘Colonic Pouches’) OR (MH ‘Ostomy’) OR (MH ‘Colostomy’) OR (MH ‘Enterostomy’) OR (MH ‘Ileostomy’)	(MH ‘Body Image’) OR (MH ‘Body Dissatisfaction’) OR (MH ‘Body Satisfaction’)	(MH ‘Attitude’) OR (MH ‘Judgement’) OR (MH ‘Emotions’) OR (MH ‘Cognition’) OR (MH ‘Perception’) OR (MH ‘Thinking’) OR (MH ‘Psychological Phenomena’) OR (MH ‘Mental Processes’)
PsychINFO (EbscoHost)	DE ‘Colostomy’	DE ‘Body Image’ OR DE ‘Body Dissatisfaction’ OR DE ‘Body Esteem’ OR DE ‘Body Image Disturbances’	DE ‘Attitudes’ OR DE ‘Cognition’ OR DE ‘Thinking’ OR DE ‘Thought Patterns’ OR DE ‘Judgement’ OR DE ‘Fear’ OR DE ‘Shame’ OR DE ‘Perception’ OR DE ‘Emotional Adjustment’ OR DE ‘Emotional Disturbances’ OR DE ‘Emotional States’ OR DE ‘Emotions’ OR DE ‘Psychosocial Outcomes’ OR DE ‘Cognitive Processes’ OR DE ‘Cognitions’ OR DE ‘Conceptual Imagery’ OR DE ‘Imagery’ OR DE ‘Schema’
CINAHL (EbscoHost)	(MH ‘Ostomy’) OR (MH ‘Ileostomy’) OR (MH ‘Colostomy’) OR (MH ‘Enterostomy’) OR (MH ‘Ostomy Care’) OR (MH ‘Ostomy Care (Iowa NIC)’) OR (MH ‘Bowel Ostomy Care (Saba CCC)’) OR (MH ‘Bladder Ostomy Care (Saba CCC)’) OR (MH ‘Surgical Stoma’)	(MH ‘Body Image’) OR (MH ‘Body Image Disturbance (NANDA)’) OR (MH ‘Body Image (Iowa NOC)’) OR (MH ‘Body Image Disturbance (Saba CCC)’) OR (MH ‘Body Dissatisfaction’) OR (MH ‘Body Satisfaction’)	(MH ‘Thinking’) OR (MH ‘Perception’) OR (MH ‘Cognition’) OR (MH ‘Mental Processes’) OR (MH ‘Psychological Processes and Principles’) OR (MH ‘Judgement’) OR (MH ‘Emotions’) OR (MH ‘Fear’) OR (MH ‘Shame’) OR (MH ‘Psychological Distress’) OR (MH ‘Affect’)

## APPENDIX B Data Extraction Form

**Table:**

Paper ID
Authors
Publication year
Location (country/setting)
Study design/methodology
Key findings (including effect sizes, where applicable)
Notes/comments (e.g., limitations, relevance, quality flags)
