# Supplementary material for: The psychological experience of body image change in adult stoma patients: A systematic review and qualitative meta‐synthesis
Source: Br J Health Psychol. 2026 Oct 12;31(4):e70113. doi: 10.1111/bjhp.70113 (PMC13658926; doi:10.1111/bjhp.70113)
Supplement: Supplementary file 1 [file BJHP-31-0-s001.docx]

**Supplementary Materials**

**Supplementary Materials A - JBI Critical Appraisal Checklist for Qualitative Research (Lockwood et al., 2015)**

|  | Yes | No | Unclear | Not applicable |
| --- | --- | --- | --- | --- |
| 1. Is there congruity between the stated philosophical perspective and the research methodology? | □ | □ | □ | □ |
| 2. Is there congruity between the research methodology and the research question or objectives? | □ | □ | □ | □ |
| 3. Is there congruity between the research methodology and the methods used to collect data? | □ | □ | □ | □ |
| 4. Is there congruity between the research methodology and the representation and analysis of data? | □ | □ | □ | □ |
| 5. Is there congruity between the research methodology and the interpretation of results? | □ | □ | □ | □ |
| 6. Is there a statement locating the researcher culturally or theoretically? | □ | □ | □ | □ |
| 7. Is the influence of the researcher on the research, and vice- versa, addressed? | □ | □ | □ | □ |
| 8. Are participants, and their voices, adequately represented? | □ | □ | □ | □ |
| 9. Is the research ethical according to current criteria or, for recent studies, and is there evidence of ethical approval by an appropriate body? | □ | □ | □ | □ |
| 10. Do the conclusions drawn in the research report flow from the analysis, or interpretation, of the data? | □ | □ | □ | □ |

Overall appraisal:	Include	□	Exclude	□	Seek further info □

Comments (Including reason for exclusion)

________________________________________________________________________________________________________________________________________________________________________________________________

Discussion of Critical Appraisal Criteria

*How to cite: Lockwood C, Munn Z, Porritt K. Qualitative research synthesis: methodological guidance for systematic reviewers utilizing meta-aggregation. Int J Evid Based Healthc. 2015;13(3):179–187.*

1. **Congruity between the stated philosophical perspective and the research methodology**

Does the report clearly state the philosophical or theoretical premises on which the study is based? Does the report clearly state the methodological approach adopted on which the study is based? Is there congruence between the two? For example:

A report may state that the study adopted a critical perspective and participatory action research methodology was followed. Here there is congruence between a critical view (focusing on knowledge arising out of critique, action and reflection) and action research (an approach that focuses on firstly working with groups to reflect on issues or practices, then considering how they could be different; then acting to create a change; and finally identifying new knowledge arising out of the action taken). However, a report may state that the study adopted an interpretive perspective and used survey methodology. Here there is incongruence between an interpretive view (focusing on knowledge arising out of studying what phenomena mean to individuals or groups) and surveys (an approach that focuses on asking standard questions to a defined study population); a report may state that the study was qualitative or used qualitative methodology (such statements do not demonstrate rigour in design) or make no statement on philosophical orientation or methodology.

2. **Congruity between the research methodology and the research question or objectives**

Is the study methodology appropriate for addressing the research question? For example:
A report may state that the research question was to seek understandings of the meaning of pain in a group of people with rheumatoid arthritis and that a phenomenological approach was taken. Here, there is congruity between this question and the methodology. A report may state that the research question was to establish the effects of counselling on the severity of pain experience and that an ethnographic approach was pursued. A question that tries to establish cause-and effect cannot be addressed by using an ethnographic approach (as ethnography sets out to develop understandings of cultural practices) and thus, this would be incongruent.

3. **Congruity between the research methodology and the methods used to collect data**

Are the data collection methods appropriate to the methodology? For example:

A report may state that the study pursued a phenomenological approach and data was collected through phenomenological interviews. There is congruence between the methodology and data collection; a report may state that the study pursued a phenomenological approach and data was collected through a postal questionnaire. There is incongruence between the methodology and data collection here as phenomenology seeks to elicit rich descriptions of the experience of a phenomena that cannot be achieved through seeking written responses to standardized questions.

4. **Congruity between the research methodology and the representation and analysis of data**

Are the data analyzed and represented in ways that are congruent with the stated methodological position? For example:

A report may state that the study pursued a phenomenological approach to explore people’s experience of grief by asking participants to describe their experiences of grief. If the text generated from asking these questions is searched to establish the meaning of grief to participants, and the meanings of all participants are included in the report findings, then this represents congruity; the same report may, however, focus only on those meanings that were common to all participants and discard single reported meanings. This would not be appropriate in phenomenological work.

5. **There is congruence between the research methodology and the interpretation of results**

Are the results interpreted in ways that are appropriate to the methodology? For example:

A report may state that the study pursued a phenomenological approach to explore people’s experience of facial disfigurement and the results are used to inform practitioners about accommodating individual differences in care. There is congruence between the methodology and this approach to interpretation; a report may state that the study pursued a phenomenological approach to explore people’s experience of facial disfigurement and the results are used to generate practice checklists for assessment. There is incongruence between the methodology and this approach to interpretation as phenomenology seeks to understand the meaning of a phenomenon for the study participants and cannot be interpreted to suggest that this can be generalized to total populations to a degree where standardized assessments will have relevance across a population.

6. **Locating the researcher culturally or theoretically**

Are the beliefs and values, and their potential influence on the study declared? For example:

The researcher plays a substantial role in the qualitative research process and it is important, in appraising evidence that is generated in this way, to know the researcher’s cultural and theoretical orientation. A high quality report will include a statement that clarifies this.

7. **Influence of the researcher on the research, and vice-versa, is addressed**

Is the potential for the researcher to influence the study and for the potential of the research process itself to influence the researcher and her/his interpretations acknowledged and addressed? For example:

Is the relationship between the researcher and the study participants addressed? Does the researcher critically examine her/his own role and potential influence during data collection? Is it reported how the researcher responded to events that arose during the study?

8. **Representation of participants and their voices**

Generally, reports should provide illustrations from the data to show the basis of their conclusions and to ensure that participants are represented in the report.

9. **Ethical approval by an appropriate body**

A statement on the ethical approval process followed should be in the report.

10. **Relationship of conclusions to analysis, or interpretation of the data**

This criterion concerns the relationship between the findings reported and the views or words of study participants. In appraising a paper, appraisers seek to satisfy themselves that the conclusions drawn by the research are based on the data collected; data being the text generated through observation, interviews or other processes.

**Supplementary Materials B – Initial Themes Found in Each Study**

| **Study** | | **Author/s (year)** | | **Initial Themes** |
| --- | --- | --- | --- | --- |
|  | 1 | | Kandemir and Oskay (2017) | - Heightened shame and anxiety - Increased acceptance over time, particularly with the support of family - Fragmented bodily identity |
|  | 2 | | Petersén and Carlsson (2021) | - Shame and anxiety - Increasing acceptance |
|  | 3 | | Reisdorfer et al. (2019) | - Mourning the premorbid body - Rejecting the stoma - Shame |
|  | 4 | | Beitz (1999) | - Social rejection - Mourning the premorbid body - Fragmented bodily self - Altered self-image |
|  | 5 | | Vural et al. (2016) | - Preserving self-worth |
|  | 6 | | Sammut et al. (2017) | - Altered self-image - Perceived discrepancy from body image standards - Rumination - Chronicity and permanency |
|  | 7 | | Cardoso et al. (2015) | - Chronic shame - Unavoidable distress - Perceived discrepancy from body image standards |
|  | 8 | | González et al. (2016) | - Loss of bodily identity - Shame - Increased acceptance over time, particularly with the support of family |
| 9 | | Tomasi et al. (2022) | | - Internalised stigma - Shame - Appearance as strange - Dual impact of ageing |
| 10 | | Mathieu et al. (2025) | | - Disidentification as a way to cope |
| 11 | | Persson and Hellström (2002) | | - Shame and disgust - Disidentification - Unexpected level of change - Acceptance - Weight loss in combination with the stoma |
| 12 | | Thorpe et al. (2016) | | - Fear of visibility to others - Loss of bodily identity - Self as unattractive - External factors influencing acceptance - Body image change as lifelong |
| 13 | | Savard and Woodgate (2009) | | - Internalised social stigma - Shame and disgust - Acceptance |
| 14 | | Smith et al. (2017) | | - Unexpected level of change - Disembodiment - Chronic and inescapable distress - Appearance as a threat |
| 15 | | Hueso-Montoro et al. (2016) | | - Utilisation of disidentification - Fear of social rejection |
| 16 | | Marques et al. (2018); | | - Body as strange - Denial of bodily change - Disidentification with the stoma |
| 17 | | Villa et al. (2018) | | - Body as disturbing |
| 18 | | Palomero-Rubio et al. (2018) | | - Internalisation of body image norms - Anxiety - Fragmented sense of self as protective |

**Supplementary Materials C – Concept Map for Each Study**

| **Study** | **Author/s (year)** | **Concepts** | | | | |
| --- | --- | --- | --- | --- | --- | --- |
|  |  | **Acceptance** | **Stigma** | **Self-Criticism** | **Emotional Distress** | **Disidentification with the body** |
| 4 | Beitz (1999) |  | “being different” | “decreased self-respect”  “changes that were threatening to the self” |  | “alienation”  “describing themselves in the third person” |
| 11 | Persson and Hellström (2002) | “once at home… more comfortable”  “lost weight… was satisfied with body” | “being different” | “not prepared”  “attractiveness... significantly decreased” | “disgust” | “alienation”  “raw meat” (when describing their feelings of disgust)  “emotional shock” (when seeing the stoma for the first time) |
| 13 | Savard and Woodgate (2009) | Acceptance enabled them “to reveal their true self to others” | “felt stigmatised” |  | “embarrassed” |  |
| 6 | Sammut et al. (2017) |  | “disconsonant from... ideal body image” | “mental picture had changed” | “significant and ongoing”  “often reflected on how she looked and what she had become” |  |
| 7 | Cardoso et al. (2015) |  | “different from other people”  “social expectations on women of the perfect body” |  | “body mark will not let the person forget”  “mechanisms to hide” | “disharmonious image” |
| 5 | Vural et al. (2016) |  |  | “feelings of attractiveness were unaffected” |  |  |
| 8 | González et al. (2016) | “related acceptance to their age or family” |  | “ugly and mutilated” | “ashamed” |  |
| 15 | Hueso-Montoro et al. (2016) | “importance of seeing as something normal”  (concerns about possible) “difficulties to get accustomed to this change” | “feel different” |  | Avoidance, mark wont let people forget, deep distress | “giving them names” (as a method to support acceptance)  “thingification” |
| 12 | Thorpe et al. (2016) | “expectation that time would bring renewed bodily strength… uncertain about when”  “familiarity facilitated acceptance”  “appearance… continued to trouble some” | “others seeing it … sought to avoid”  “dressed to disguise”  “less like a woman” | “deeply unattractive”  “disfigured” | “shock and horror”  “deeply distressing” | “bodies as unfamiliar” |
| 1 | Kandemir and Oskay (2017) | “began not to feel uncomfortable” (around family) |  |  | “avoided eye contact”  “disturbed by the opening” | “loss of integrity” |
| 14 | Smith et al. (2017) | “desensitised” | “imperfect ‘other’ rather than complete ‘perfect’ woman” | “exaggerating the contrast between before and after”  “perception of being older”  “nasty shock”  “new relationship with body … more open and less private” | “debilitating”  “psychologically disturbing”  “horror” | “alien force”  “internal … on the outside of the body”  “unnatural”  “sense of objectification and depersonalisation”  “separate from her physical self” |
| 16 | Marques et al. (2018); | “humorous” (when discussing methods of acceptance of bodily change)  “apparent acceptance … dissonances merge” (acceptance of disgust) |  | “strangeness”  “denial” |  | “’a thing’”  “put there”  “artefact … even when the person … seems to accept” |
| 17 | Villa et al. (2018) | “did not change the way they saw themselves” |  | “disturbed … aesthetically” |  |  |
| 18 | Palomero-Rubio et al. (2018) | (able to reach acceptance because the) “body is seen as … external” | “body differs from the canons of … beauty laid down by society”  “social declassification”  “socially disapproved” |  | “anger”  “anxiety”  “fear”  “anguish” | “divided and split” |
| 3 | Reisdorfer et al. (2019) |  | “women feel… less attractive” |  | “seek to disguise”  “preferring death” | “feeling incomplete” |
| 2 | Petersén and Carlsson (2021) | “after a while… bought clothes they liked” | “viewed with disgust” (in public places) |  | “hide their stomas”  “ashamed” |  |
| 9 | Tomasi et al. (2022) |  | “different person” (to others) | “strange body” | “embarrassed” |  |
| 10 | Mathieu et al. (2025) |  |  |  |  | “coped by personifying”  “perceiving as external lessened threat to… identity” |

**Supplementary Materials D – GRADE-CERQual Evidence Profile**

| **Review Finding** | **GRADE-CERQual Confidence** | **Explanation** |
| --- | --- | --- |
| All-consuming shock | Moderate | Moderate concerns were found regarding methodological limitations (the influence of the researcher’s position was not stated in 15 studies; however, appropriate application of methodology was found to be consistent). Minor concerns were noted regarding coherence, with unexplained inconsistencies between findings in one study, and otherwise consistent patterns in how all-consuming shock was explained. Moderate concerns were identified regarding adequacy because two of the six contributing studies provided limited richness and quantity of data supporting this finding. Relevance was strong across all studies, with all studies focussing on the psychological experience of stoma surgery in adult populations. |
| Visible ‘Otherness’ | Moderate | Moderate concerns were found for methodological limitations, particularly with regards to reflexivity, and consistency between the philosophical/ methodical positionings and methods used. Some contradictory data was not accounted for across studies, but ‘visible otherness’ appears clearly and repeatedly and was well grounded within the raw data, meaning concerns with coherence were low. There were low concerns about adequacy, with ample and rich data supporting this finding across all of the included studies. Findings are directly applicable to the review question and its associated context, therefore there were no concerns with relevance. Overall confidence was rated moderate because the methodological limitations, particularly limited reflexivity and epistemological transparency, may have influenced the interpretation of the finding. |
| Fluctuating acceptance | Moderate | Moderate concerns regarding methodological limitations (8 studies showed moderate limitations, including lack of reflexivity, triangulation, and epistemological consistency and/or transparency). There were low concerns about coherence, with findings demonstrating consistent and well-integrated patterns across studies. Moderate concerns regarding adequacy were identified due to limited richness and quantity of data supporting this finding in 6 studies. There are no concerns regarding relevance; the included findings directly reflect the population, setting, and phenomenon of interest in the review question. |
